# Differential Role of Cathepsins S and B In Hepatic APC-Mediated NKT Cell Activation and Cytokine Secretion

**DOI:** 10.3389/fimmu.2018.00391

**Published:** 2018-02-28

**Authors:** Álvaro de Mingo Pulido, Estefanía de Gregorio, Shilpi Chandra, Anna Colell, Albert Morales, Mitchell Kronenberg, Montserrat Marí

**Affiliations:** ^1^Department of Cell Death and Proliferation, Institut d’Investigacions Biomèdiques de Barcelona (IIBB-CSIC) and Institut d’Investigacions Biomèdiques August Pi i Sunyer (IDIBAPS), Barcelona, Spain; ^2^La Jolla Institute for Allergy and Immunology, La Jolla, CA, United States

**Keywords:** iNKT, cathepsins, inflammation, liver damage, NF-κB

## Abstract

Natural killer T (NKT) cells exhibit a specific tissue distribution, displaying the liver the highest NKT/conventional T cell ratio. Upon antigen stimulation, NKT cells secrete Th1 cytokines, including interferon γ (IFNγ), and Th2 cytokines, including IL-4 that recruit and activate other innate immune cells to exacerbate inflammatory responses in the liver. Cysteine cathepsins control hepatic inflammation by regulating κB-dependent gene expression. However, the contribution of cysteine cathepsins other than Cathepsin S to NKT cell activation has remained largely unexplored. Here we report that cysteine cathepsins, cathepsin B (CTSB) and cathepsin S (CTSS), regulate different aspects of NKT cell activation. Inhibition of CTSB or CTSS reduced hepatic NKT cell expansion in a mouse model after LPS challenge. By contrast, only CTSS inhibition reduced IFNγ and IL-4 secretion after *in vivo* α-GalCer administration. Accordingly, *in vitro* studies reveal that only CTSS was able to control α-GalCer-dependent loading in antigen-presenting cells (APCs), probably due to altered endolysosomal protein degradation. In summary, our study discloses the participation of cysteine cathepsins, CTSB and CTSS, in the activation of NKT cells *in vivo* and *in vitro*.

## Introduction

Recent studies indicate that cysteine cathepsins serve as regulatory enzymes, acting beyond simple housekeeping proteases, that harbor important functions outside the lysosome (1). Multiple divergent roles for different cathepsins in a variety of physiologic and pathophysiologic processes have been reported (1, 2). In particular, we have recently revealed the participation of cysteine cathepsins [Cathepsin B (CTSB) and Cathepsin S (CTSS)] in controlling hepatic NF-κB-dependent inflammation *via* sirtuin-1 regulation (3), and the role of CTSB in promoting hepatic stellate cell (HSC) activation and liver fibrosis (4). Of interest, cysteine cathepsins have also been implicated in antigen presentation, being CTSS the protease most highly expressed in professional antigen-presenting cells (APCs) (5, 6).

Natural killer T (NKT) cells are unconventional T cells that express both T cell receptors (TCRs) and natural killer (NK) cell receptors. Based on TCR expression, NKT cells can be divided into classical NKT cells, also known as type I or invariant NKT cells (iNKT cells) and non-classical NKT cells or type II NKT cells. α-galactosylceramide (α-GalCer) is widely used as the model antigen to investigate iNKT cell function, where non-classical MHC class I molecule CD1d presents α-GalCer and related glycolipid antigens to iNKT cells (7–9). While synthetic and microbial antigens for iNKT cells have been defined, the nature of the self-antigens involved in the development and maturation of iNKT cells is controversial. iNKT cells have been reported to regulate a variety of immune responses, including the response to cancers and the development of autoimmunity (10). iNKT cells also represent a subset of innate-like T lymphocytes that function as orchestrators of the hepatic inflammation underpinning liver damage. In fact, the hepatic influx of activated CD8^+^ T cells and of NKT cells has been recently linked to the progression of non-alcoholic fatty liver disease to non-alcoholic steatohepatitis (NASH) and subsequently to hepatocellular carcinoma in experimental models and in patients (11).

The liver contains the highest ratio of iNKT cells/conventional T cells compared to other organs. Mouse iNKT cells account for as much as 40% of the resident, intrahepatic lymphocyte pool (12–14). In humans, however, the frequency of iNKT cells is much lower, and highly variable among individuals, ranging from 0.05% to over 1% (15–17). Upon antigen stimulation, using either the synthetic CD1d ligand α-GalCer or other CD1d-dependent antigens, iNKT cells secrete both Th1 cytokines, including interferon γ (IFNγ) and interleukin (IL)-2, and Th2 cytokines, including IL-4 and IL-13, that recruit and activate other innate immune cells to exacerbate inflammatory responses in the liver. Moreover, iNKT cells can directly cause liver injury by a Fas/Fas ligand (FasL)-dependent mechanism (18, 19), and emerging evidence supports a central role for iNKT cells in hepatic immune homeostasis and disease pathogenesis (20).

Antigen presentation by both MHC class II molecules and the non-classical MHC class I-like molecule CD1d requires entry of these proteins into the endosomal/lysosomal compartments of antigen-presenting cells (APCs) (6). In the liver, diverse cell populations can act as APCs, including Kupffer cells (KCs), liver sinusoidal endothelial cells (LSECs), hepatocytes, dendritic cells (DCs), B cells and HSCs, which all can interact with NKT cells (7, 21).

The lysosomal cysteine proteases, in particular CTSS and cathepsin L, have an important role in regulating antigen presentation by both MHC class II molecules and CD1d (6, 22, 23). In particular, CTSS was implicated in the CD1d presentation pathway by several reports describing a role for CTSS in the degradation of the class II-associated invariant chain (Ii), which can introduce CD1d into endosomal compartments. In the absence of CTSS activity, the Ii-p10 fragment is retained (5, 24–29) resulting in endosomal enlargement and probably affecting the loading of CD1d with antigenic lipids (28). In agreement, CTSS-deficient mice had decreased numbers of iNKT cells, and DCs isolated from these mice induced inefficient *in vitro* stimulation of Vα14^+^NK1.1^+^ T-cell hybridomas (30). Moreover, regarding the participation of Ii and CTSS in the thymic development of iNKT cells, a requirement for invariant chain Ii, but not for CTSS, has been reported. Ii^−/−^ mice display a reduction in thymic iNKT cells but CTSS^−/−^ mice developed iNKT cells in normal ratios (31). However, upon maturation both splenic Ii^−/−^ and CTSS^−/−^ cells produced normal levels of IFNγ but significant lower amounts of TNF (31), thus indicating a role for CTSS in the effector function of iNKT cells. Similarly, in Mycobacterium tuberculosis infection, pathogen responsible for most cases of tuberculosis worldwide, Ii presence in the infected macrophages is critical for the induction of CD1d-restricted iNKT cell response (31, 32). On the other hand, CTSS-deficient macrophages can activate iNKT cells during *Mycobacterium tuberculosis* infection (31, 32), thus indicating that this protease is dispensable in this setting. Given the ubiquitous nature of CTSB, the highly expression of CTSS observed in APCs, and our recent study reporting the participation of these two cathepsins in the regulation of hepatic inflammation (3), we aim to determine if these cathepsins could also participate in iNKT cell-derived inflammation in the liver. Therefore, we have analyzed how CTSB or CTSS inhibition impacted iNKT cell expansion and/or activation in experimental models of liver injury.

## Materials and Methods

### *In Vivo* iNKT Cell Activation in Mice

8- to 12-week old C57BL/6 male mice were obtained from Charles River. All animals received humane care according to the criteria outlined in the “Guide for the Care and Use of Laboratory Animals.” Animal procedures were approved by the Animal Experimentation Ethics Committee (CEEA) from the Universitat de Barcelona. All mice were located in the animal facilities in the Faculty of Medicine (Universitat de Barcelona), under a 12 h light/12 h dark cycle. Food and water were provided *ad libitum* unless otherwise stated. α-GalCer (Abcam) was suspended in sterile saline. Mice were injected i.p. first with vehicle, CTSB inhibitor (CA-074-Methyl, 10 mg/Kg) (33) or CTSS inhibitor (Z-FL-COCHO, 10 mg/Kg) (34) followed, 1 h later, by saline, LPS (1 mg/Kg) or 2.5 μg/mouse α-GalCer. Blood was collected after 2 h for IL-4 ELISA measurement and at 24 h for IFNγ ELISA measurement. Mice were sacrificed at the specified time-points for analysis.

### Murine iNKT Cell Analysis

Liver iNKT cells were isolated by liver perfusion with ice-cold PBS followed by mechanical disruption on a Petri dish. Briefly, the homogenate was pushed through a 75 µm cell strainer, resuspended in 50 ml RPMI-1640 media, centrifuged at 60 × *g*, 4 min, 4°C, and 45 ml of supernatant were recovered. Non-parenchymal cells were pelleted at 600 g, 4°C, 8 min and resuspended in 10 ml 37.5% Percoll followed by centrifugation at 850 × *g*, 4°C, 30 min in “break off” mode. Recovered cells were resuspended in 2 ml red blood cell lysis buffer and incubated for 5 min at RT, then 1 ml of RPMI with 10% FBS was added and cells were centrifuged at 480 × *g*, 8 min, 4°C, and resuspended in RPMI with 10% FBS. iNKT cells were identified by flow cytometry analysis.

### Flow Cytometry

Cells were counted in a Neubauer chamber and concentration adjusted to 5 × 10^5^ cells/mL. Antibodies were added in 500 µl of wash buffer with 10% rabbit serum to block nonspecific binding and incubated 30 min at 4°C while protected from light. Cells were washed twice with wash buffer (#554723, BD Biosciences) and then incubated in 300 µl BD CellFIX 1x buffer (#340181, BD Biosciences) for 15 min at 4°C. A lymphocyte gate was initially set on a forward-scatter vs side-scatter dot plot. CD4, CD8, F4/80, CD11b, and NK1.1 were used to exclude monocytes, B cells, and other non-T cells. iNKT cell population was determined as CD3-positive, CD1d-PBS-57-positive, and CD19-negative. We used anti-CD19 antibody to exclude non-specifically stained B cells, which share forward and side scatter characteristics similar to iNKT cells. CD69 was used as an activation marker for hepatic iNKT cells and AnnexinV-APC labeling kit to determine iNKT apoptosis. Antibodies and Annexin V were acquired from BD-Biosciences (San Diego, CA, USA). We used 2 µl of either CD3-FITC or mCD1d-PBS-57-PE antibody per 10^6^ cells. For the rest of antibodies 1μl/10^6^ cells was employed. Multicolor-FACS staining was performed for analysis in a BD LSRFortessa cytometer. A total of 1 × 10^6^ cells were stained in a total volume of 100 µL of FACS Binding Buffer (PBS + 2%FBS + 0.05% NaN_3_). Cell surface antibodies were incubated for 30 min at 4°C in FACS. Annexin V-APC was stained afterward after washing, following the manufacturer’s protocol, and samples were analyzed before 60 min. Data were analyzed by using Flowing Software 2 (University of Turku, Finland). mCD1d-PBS-57-PE labeled tetramers were obtained through the NIH Tetramer Core facility at Emory University (Atlanta, GA, USA).

### Isolation of Mouse Peritoneal Macrophages (PMs)

Male C57BL6/J mice aged 8–12 weeks were treated with 3% thioglycollate broth (Sigma-Aldrich) 4 days before they were sacrificed. PMs were isolated by lavage using ice-cold Ca^2+^ and Mg^2+^-free PBS and plated in 6-well plates at a density of 10^6^ cells per well in complete RPMI medium.

### KC Isolation

C57BL/6 male mice, 8–12 weeks old, were from Charles River (Wilmington, MA, USA). KCs were isolated by liver perfusion with collagenase-pronase as described (3) with small modifications. Hepatocytes were separated from non-parenchymal cells by 60 × *g* centrifugation. The supernatant containing non-parenchymal cells was collected and centrifuged at 600 × *g* for 8 min. The non-parenchymal cell pellet was resuspended in 6 ml of GBSS (Gey’s Balanced Salt Solution) and mixed with 6 ml of cold Nycodenz at 32% to reach 16%, then it was topped with 2 ml GBSS and centrifuged at 2,300 × *g*, 45 min, 4°C without break. KCs appear in the interface, were collected with a Pasteur tip, washed twice with GBSS at 500 g, 5 min, 4°C and resuspended in RPMI complemented with 10% FBS, and antibiotics at 37°C in a humidified atmosphere of 95% air and 5% CO_2_.

### Nuclear Extract Preparation

2 × 10^6^ cells were scraped in Buffer A [10 mmol/L Hepes, 10 mmol/L KCl, 0.1 mmol/L ethylenediaminetetraacetic acid, 0.1 mmol/L ethylene glycol tetraacetic acid (EGTA), 1 mmol/l dithiothreitol (DTT), and 0.5 mmol/l phenylmethylsulfonyl fluoride (PMSF)], kept on ice for 15 min, lysed by the addition of 1/20 (vol/vol) 10% Igepal and vortexed for 10 s. Nuclei were pelleted (12,000 g, 30 s), resuspended in Buffer C (20 mmol/l Hepes, 0.4 mol/l NaCl, 1 mmol/l EDTA, 1 mmol/l EGTA, 1 mmol/l DTT, and 1 mmol/l PMSF), and incubated for 15 min on ice with gentle mixing. Subsequently, nuclear extracts were obtained by centrifuging at 4°C, 12,000 × *g* for 5 min.

### Antigen Presentation Assay

Antigen-presenting cells were loaded with antigen (100 ng/ml α-GalCer) in a slow shaker during a 6 h at incubation. iNKT cells were thawed at least 2 days before each experiment and cultured in RPMI complete medium supplemented with IL-2 (10 U/mL) for up to 7 days. We cultured 50,000 APCs and 50,000 iNKT cells per well in a 24-well plate. The plates were spun 1 min for better interaction, supernatants collected at 24 h for IFNγ determination, and cellular pellets were stained with 0.2% Trypan Blue to assess cathepsin inhibitor toxicity.

Co-culture of KCs and iNKTs. KCs isolated and cultured in RPMI + 10%FBS. The day after KCs were changed to RPMI + 2% FBS and incubated with LPS (50 ng/ml) for 6 h with or without preincubation with CA-074 (75 µM) or CTSS (7.5 µM) for 1 h. iNKT cells were isolated by cell sorting and seeded on top of KCs with or without CA-074 (75 µM) or CTSS (7.5 µM). We cultured 50,000 KCs and 50,000 iNKT cells per well in a 24-well plate. The plates were spun 1 min for better interaction, supernatants collected at 24 h for IFNγ determination, and cellular pellets were stained with 0.2% Trypan Blue to assess cathepsin inhibitor toxicity.

### Cell Lines and Treatment

LX2 cells were kindly given by Dr. Ramón Bataller (IDIBAPS, Barcelona), and APCs C1R-CD1d cells were described previously (35). LX2 cells were maintained in DMEM, while C1R-CD1d and RAW264.7 cells were cultured in RPMI. Medium was supplemented with 10% heat-inactivated FBS, penicillin (100 U/mL), and streptomycin (100 µg/mL, Invitrogen). α-GalCer (Abcam) was given to cells at 100 ng/mL, for 6 h. CTSB inhibitor (75 µM, CA-074 methyl ester, Sigma-Aldrich) and CTSS inhibitor (7.5 µM, Z-FL-COCHO, Calbiochem) were administered 1 h before α-GalCer.

### Human iNKT Cell Isolation

Human Vα24*i* NKT cell lines were generated using blood with modifications to a published protocol (36). Informed consent was obtained from all subjects. Subjects include healthy male and female subjects aged 20–40. Blood sample was obtained using standard phlebotomy techniques. Study was approved by LA Jolla Institute for Allergy and Immunology Institutional Review Board. PBMCs were isolated from the blood and were stimulated with 100 ng/ml α-GalCer (Kyoka Hakko Kirin Co., Ltd.). Next day recombinant human IL-2 (Biolegend) was added at a concentration of 30 ng/ml. After 10–15 days, cells were sorted using 6b11 (Becton Dickinson) antibody. Cells thus obtained were restimulated with PBMCs loaded with αGalCer. For loading, PBMCs were pulsed with 100 ng/mL of α-GalCer for 4–5 h before irradiation at 37°C. Cells were further irradiated with X-ray equivalent for 3,000 rads. Washed cells were added to sorted NKT cells 1NKT:5PBMCs ratio. IL-2 was added next day at a concentration of 30 ng/ml. Cells were expanded for next 10–14 days.

### Mouse iNKT Cell Isolation

Primary mouse iNKT cells were obtained from livers of C57BL/6J mice (*n* = 3). Cells were stained with LIVE/DEAD™ Fixable Violet Dead Cell Stain Kit (L34955, Life Technologies) to exclude dead cells. The sorting was performed selecting CD3-FITC^+^, mCD1d-PBS-57-PE^+^ iNKT cells in a BD FACSAria III sorter at the IDIBAPS Cell Sorting Facility.

### SDS-PAGE and Immunoblot Analysis

Cell lysates were prepared in RIPA buffer (50 mM Tris·HCl, pH 8, 150 mM NaCl, 1% Nonidet P-40, 0.1% SDS, 1% Triton X-100 plus proteinase inhibitors). Protein concentration was determined by Bradford assay, and samples containing 10–50 µg were separated by SDS-PAGE. Proteins were transferred to nitrocellulose membranes. After, membranes were blocked in 8% nonfat milk in 20 mM Tris–HCl, 150 mM NaCl, and 0.05% Tween-20 for 1 h at room temperature. Antibodies were used 2 h at room temperature for immunoblotting, including anti-NPC2 (sc-33776), anti-CD1d (sc-19632), anti-CD74 (sc-47742), anti-p65-NF-κB (sc-372), anti-Lamin (sc-6215), anti-pro-saposin (Biorbyt, orb13663), or anti-β-actin-HRP (A3854, Sigma, 30 min, room temperature).

### Tissue Analyses

Livers were formalin-fixed and 7-µm sections were routinely stained with H&E following standard procedures. The slices were examined with a Zeiss Axioplan microscope equipped with a Nikon DXM1200F digital camera.

### Enzyme-Linked Immunosorbent Assay (ELISA)

96-well ELISA plates were coated with 100 μL/well purified capture antibody specific for IL-4 or IFNγ that were diluted in PBS at 1:250. The ELISA plates were washed and processed according to standard protocols.

### RNA Isolation and Real-time RT-PCR

Total RNA from primary KCs was isolated with TRIzol reagent. Real-time RT-PCR was performed with iScript™ One-Step reverse transcription–PCR Kit with SYBR^®^ Green following the manufacturer’s instructions (BioRad). The sequences of the primers used are as follows: m-IL12-FW, 3′-GGAAGCACGGCAGCAGAATA-5′; m-IL12-RV, 3′-AACTTGAGGGAGAAGTAGGAATGG-5′; m-b-Actin-FW, 3′- GACGGCCAGGTCATCACTAT-5′; m-b-Actin-RV, 3′- CGGATGTCAACGTCACACTT-5′.

### Transaminases

Alanine and aspartate transaminases (ALT and AST) in sera were measured using a biochemical analyzer at Clinic Hospital, Barcelona.

### Statistical Analysis

All *in vitro* experiments were repeated at least three times. Results are expressed as mean ± SD for cell studies, and as mean ± SEM for *in vivo* studies. Statistical comparisons were performed using unpaired two-tailed Student’s *t*-test. All analyses were performed using GraphPad Prism. A *P*–value <0.05 was considered significant.

## Results

### CTSB and CTSS Inhibitors Reduce iNKT Cell Activation during LPS-Induced Inflammation

**Figure 1:**
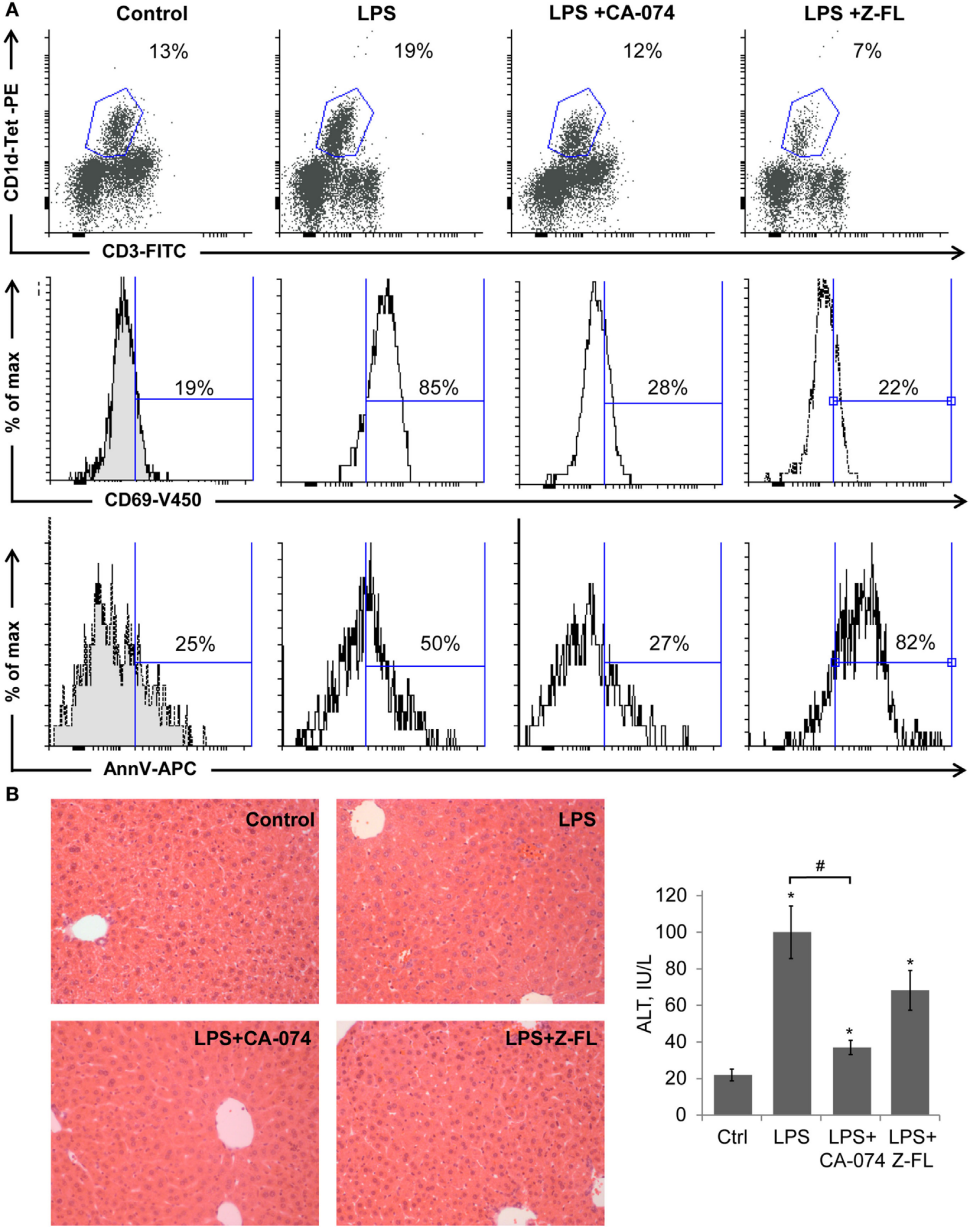
Cathepsin B (CTSB) and Cathepsin S (CTSS) inhibitors reduce iNKT activation after LPS challenge. Mice were treated with CTSB or CTSS inhibitors (CA-074 or Z-FL, 10 mg/kg, i.p., respectively) 1 h before LPS injection (1 mg/kg). **(A)** Liver iNKT cells were isolated by liver perfusion, determined by FACS, and activation marker CD69 and apoptotic cell death by Annexin V were determined. **(B)** H&E staining (magnification 20×) of liver samples and liver damage determined by ALT values. *p < 0.05 vs Control, and ^#^*p* < 0.05 vs LPS. Representive images of three independent experiments (*n* = 3, each experiment).

It has been demonstrated that LPS activates iNKT cells in the absence of TCR engagement by a foreign Ag, by integrating two types of signals: (a) cytokines mediated, IL-12 particularly, from TLR4-stimulated APCs (37) and (b) by TCR-mediated recognition of induced self-Ag (38). The activated iNKT cells then rapidly amplify innate immune responses initiated by APCs. Given the importance of LPS as a central player in inducing inflammation in multiple liver diseases, we evaluated if iNKT cell activation in response to LPS is influenced by cysteine cathepsins. To this aim, we administered LPS to mice in the absence or presence of CTSB (33) or CTSS (34) specific inhibitors (CA-074-Methylester and Z-FL-COCHO, respectively) and analyzed the iNKT cell population by flow cytometry analysis by selecting double positive CD3^+^ and CD1d-PBS-57 tetramer^+^ (CD1d-Tet^+^) cells. We observed a basal population of iNKT cells, with an average of approximately 13% of the total found in control mice. This was increased after LPS treatment (Figure 1A). The CTSB inhibitor blocked this iNKT cell increase, and interestingly, the CTSS inhibitor not only blocked the increase, but caused a decrease in the frequency of the iNKT cell population to approximately 7% (Figure 1A). These results indicate that CTSB and CTSS influence the extent of iNKT cell activation leading to a decrease in this population. When analyzing CD69, as an activation marker for iNKTs, we observed that only LPS-treated animals display a significant increase (85 vs 19% in control samples) in the iNKT population. This activation was prevented by CTSB and CTSS inhibitors, indicating that these cathepsins participate in the activation of hepatic iNKT cells after LPS challenge (Figure 1A).

LPS-induced moderate apoptosis of activated hepatic iNKT cells, as detected by Annexin V labeling, indicating that there is activation followed by cell death under these stimuli. Of note, CTSS inhibition resulted in enhanced cell death of hepatic iNKT cells without patent activation (Figure 1A).

H&E staining in Figure 1B showed that LPS resulted in modest inflammatory cell recruitment and hepatic damage, confirmed by measuring serum transaminase values. CTSB inhibition not only blocked iNKT cell expansion but also reduced liver damage, consistent with the specific role of CTSB in TNF-dependent apoptotic pathways in hepatocytes (39). By contrast, the CTSS inhibitor did not significantly alter hepatic damage despite the fact that iNKT activation was clearly prevented. This last observation probably reflects the fact that hepatic damage after LPS challenge is not exclusively dependent on iNKT cell activation, since many other cell types respond to LPS by inducing cytokine secretion and causing hepatic damage (40).

**Figure 2:**
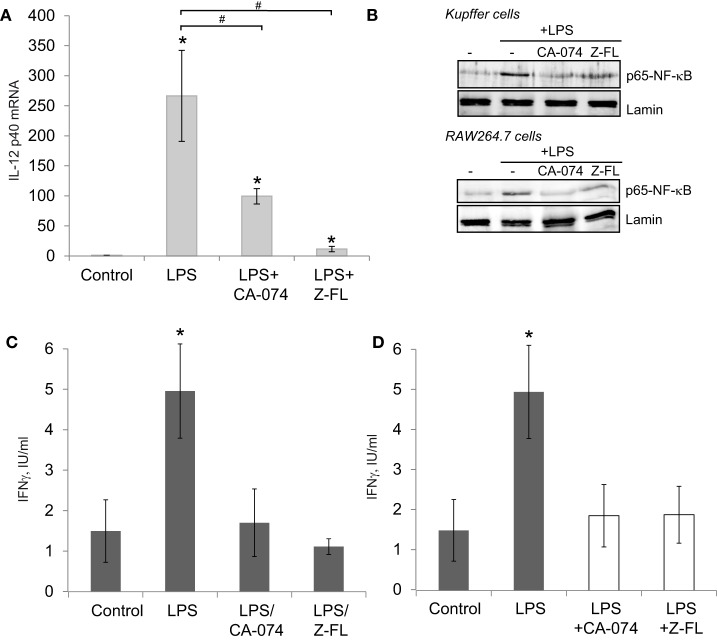
LPS-dependent IL-12 mRNA expression in Kupffer cells (KCs) and iNKT cell activation is decreased after Cathepsin B (CTSB) or Cathepsin S (CTSS) inhibition. **(A)** IL-12 mRNA expression in primary KCs treated with CTSB (CA-074, 75 µM) or CTSS inhibitor (Z-FL, 7.5 µM) for 1 h before LPS challenge (50 ng/mL, 4 h). **(B)** KCs and RAW264.7 cells were pre-incubated with CA-074 (75 µM) or Z-FL (7.5 µM) for 1 h and nuclear proteins were analyzed after LPS (50 ng/ml, 30 min) challenge. Lamin was used as nuclear loading control. **(C)** KCs were preincubated with or without CTSB and CTSS inhibitors (75 and 7.5 µM, respectively, for 1 h) before being treated with LPS (50 ng/mL, 6 h). Afterward, KCs were washed and co-cultured with mouse iNKT cells, and IFNγ was determined in the extracellular media by enzyme-linked immunosorbent assay (ELISA) after 24 h. **(D)** KCs were treated with LPS (50 ng/mL, 6 h). Afterward, KCs were co-cultured the with mouse iNKT cells in the presence or absence of CTSB or CTSS inhibitors and IFNγ was determined in the extracellular media by ELISA after 24 h. *p < 0.05 vs Control, and ^#^*p* < 0.05 vs LPS.

Since IL-12 production by TLR4-stimulated APCs can activate iNKT cells independently of the TCR (37), we determined IL-12 expression in primary Kuppfer cells, hepatic resident macrophages, exposed to LPS, and the effect of cathepsin inhibitors. KCs responded to LPS by inducing IL-12 mRNA expression, which was significantly reduced upon CTSB or CTSS inhibition (Figure 2A). KCs in the liver are the main APCs responsible for IL-12 secretion after bacterial infection (41), and LPS-derived IL-12 production in macrophages is an NF-kB-dependent event (42). Thus, we determined if CTSB or CTSS inhibition affected p65-NF-kB nuclear translocation in primary isolated KCs and also in murine RAW264.7 cell macrophages. As previously reported (3), LPS-induced translocation of the active p65-NF-kB subunit to the nucleus in KCs and RAW264.7 cells was prevented by CTSB or CTSS inhibitors (Figure 2B).

Since hepatic iNKT activation by LPS requires two steps, first KC-dependent generation of IL-12 and second IL-12-dependent iNKT activation and IFNγ secretion by iNKT cells, we performed *in vitro* co-culture experiments with primary mouse KCs and iNKT cells using two approaches to further investigate the role of CTSB and CTSS inhibitors in this model. In the first approach, and to evaluate to role of the inhibitors on KCs, KCs were incubated first with CTSB or CTSS inhibitors for 1 h, and then cells were thoroughly washed and incubated with LPS for 6 h followed by addition of mouse iNKT cells. IFNγ was detected in the culture media 24 h afterward (Figure 2C). IFNγ increased after LPS challenge but was significantly decreased upon CTSB or CTSS inhibition. These results are consistent with the decreased LPS-dependent IL-12 generation detected in KCs upon CTSB or CTSS inhibition (Figure 2A).

In the second approach, to evaluate the role of the inhibitors on iNKT cells, KCs were incubated alone with LPS for 6 h, followed by addition of iNKT cells along with CTSB and CTSS inhibitors. IFNγ was detected in the culture media 24 h afterward (Figure 2D). Under these conditions, IFNγ was similarly enhanced after LPS challenge and again CTSB and CTSS inhibitors decreased IFNγ secretion in iNKT cells. This observation is consistent with a direct role for CTSB and CTSS on iNKT-dependent IFNγ generation.

### CTSS Inhibitors Reduce α-GalCer-Induced Hepatitis

**Figure 3:**
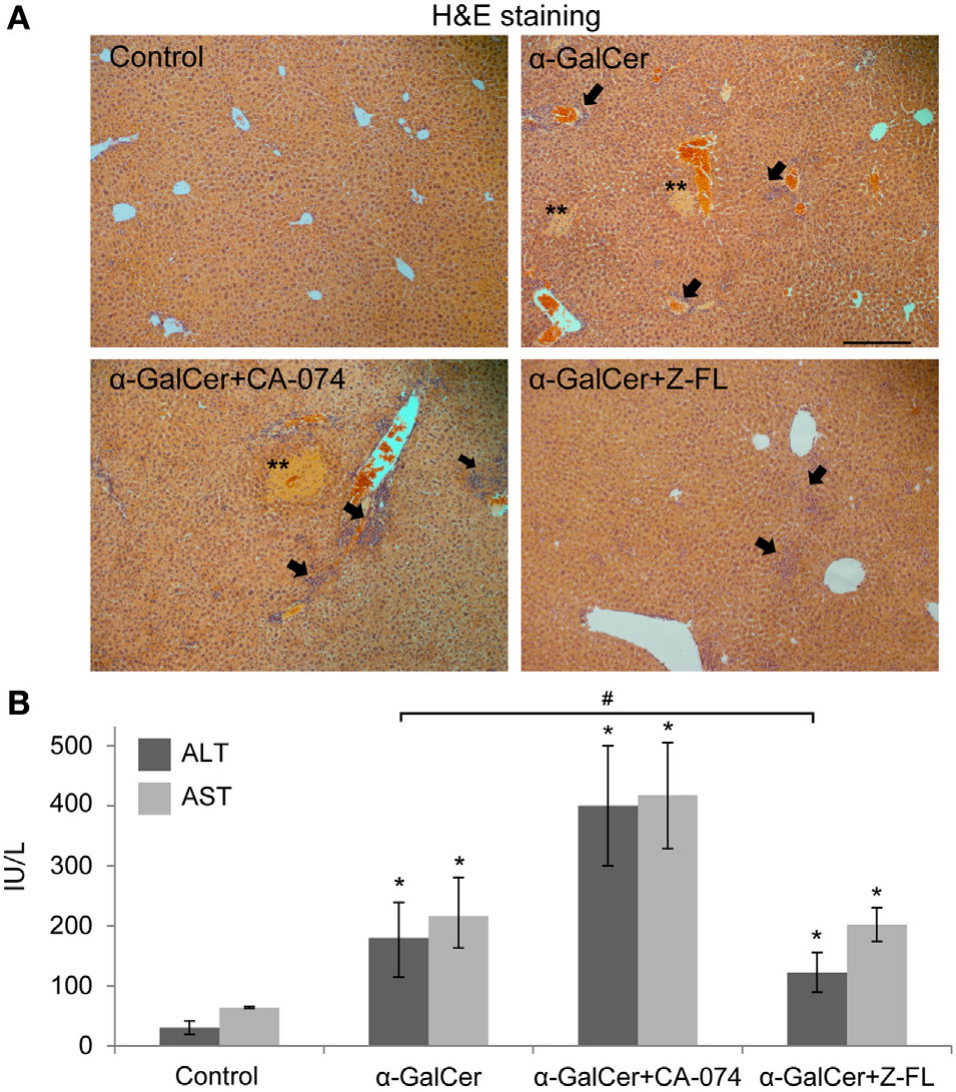
Effect of Cathepsin B (CTSB) and Cathepsin S (CTSS) inhibitors in liver damage after α-GalCer challenge. Mice were treated with CTSB or CTSS inhibitors (CA-074 or Z-FL, 10 mg/kg, i.p., respectively) 1 h before α-GalCer (2.5 µg/mouse) injection. **(A)** H&E staining of liver samples, arrows indicate inflammatory foci (magnification 20×). Representive images of three independent experiments (*n* = 5, each experiment). **(B)** Liver damage determined by ALT and AST values. *p < 0.05 vs Control and ^#^*p* < 0.05 vs α-GalCer. Representive images of three independent experiments (*n* = 3, each experiment).

It has been reported that activation of hepatic iNKT cells triggers hepatic injury following the administration of the potent iNKT cell specific antigen, α-GalCer (14, 43). Thus, to evaluate the direct role of cysteine cathepsin inhibitors on liver injury derived from iNKT cell activation, we treated mice with CTSB or CTSS inhibitors 1 h prior α-GalCer administration. 24 h after α-GalCer challenge, there was evident liver damage accompanied by inflammation and the presence of some necrotic areas, as shown by H&E staining (Figure 3A). Liver damage correlated with elevated transaminase levels (Figure 3B).

**Figure 4:**
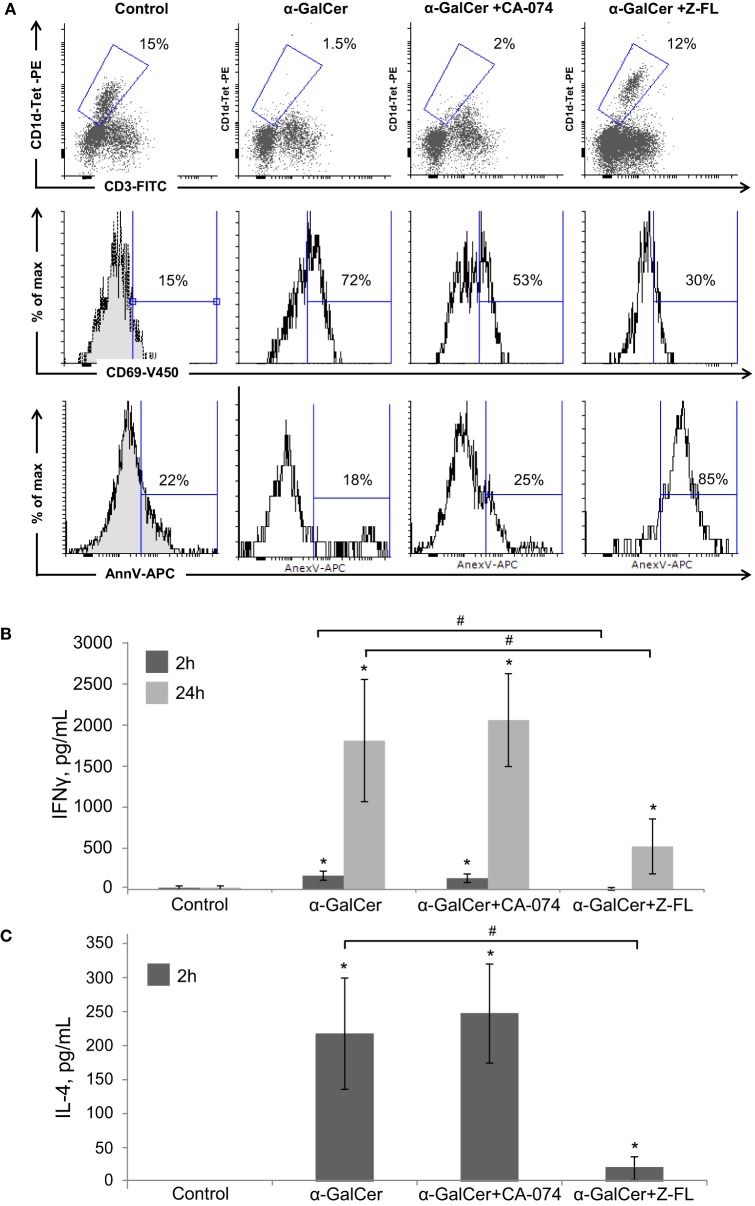
Effect of Cathepsin B (CTSB) and Cathepsin S (CTSS) inhibitors in NKT activation and cytokine secretion after α-GalCer challenge. Mice were treated with CTSB or CTSS inhibitors (CA-074 or Z-FL, 10 mg/kg, i.p., respectively) 1 h before α-GalCer (2.5 µg/mouse) injection. **(A)** Liver iNKT cells were isolated by liver perfusion, determined by FACS, and activation marker CD69 and apoptotic cell death by Annexin V were determined. **(B,C)** IFNγ and IL-4 determined by enzyme-linked immunosorbent assay in peripheral blood at the indicated time points. *p < 0.05 vs Control and ^#^*p* < 0.05 vs α-GalCer. Representive images of three independent experiments (*n* = 3, each experiment).

When analyzing the activation state of iNKT cells under these conditions, we observed that iNKT cells almost disappeared upon 24 h α-GalCer challenge (Figure 4A). Furthermore, the residual hepatic iNKT cell population was mostly CD69^+^ but Annexin V negative indicating that α-GalCer did not significantly enhance iNKT cell apoptosis, consistent with previous studies (42). CTSB inhibition did not significantly affect α-GalCer-dependent iNKT cell disappearance or activation. However, CTSS inhibition prevented iNKT cell disappearance after α-GalCer challenge, since hepatic iNKT were present in normal numbers, with a moderate presence of CD69 positive iNKT cells. Of note, and similarly to what we observed after LPS challenge (Figure 1A), CTSS inhibition enhanced iNKT cell apoptosis, without activation of this cell type, indicating probably a direct effect of CTSS inhibitors on iNKT cell viability at the dose given *in vivo*.

Consistent with the activation state of iNKT cells after α-GalCer challenge, there was an increase *in vivo* in serum IFNγ at 2 h and 24 h, and in IL-4 at 2 h (Figures 4B,C) that was significantly reduced by CTSS inhibition, but not after CTSB inhibition. Similarly, CTSS inhibition, but not CTSB, resulted in decreased liver damage, as observed by H&E staining, as well as decreased ALT levels (Figures 3A,B). Thus, in the α-GalCer model the activation of iNKT cells and IFNγ generation correlate with liver damage, and are prevented by CTSS inhibition.

To further elucidate whether cysteine cathepsins specifically affect iNKT directly or have an effect on the loading of the α-Galcer antigen onto hepatic APCs we moved to *in vitro* assays.

### *In Vitro* Activation of iNKT Is Modulated by Cysteine Cathepsins

**Figure 5:**
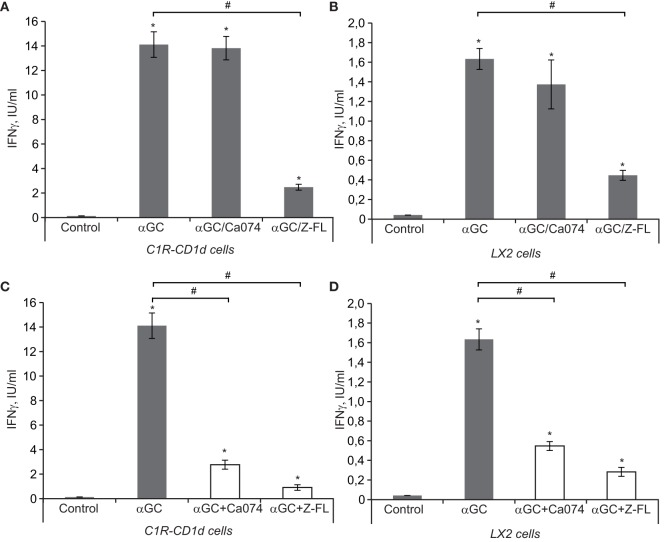
IFNγ release after iNKT activation *in vitro*. **(A,B)** antigen-presenting cells (APCs) (LX2 and C1R-CD1d) were preincubated with or without Cathepsin B (CTSB) and Cathepsin S (CTSS) inhibitors (75 and 7.5 µM, respectively, for 1 h) before being treated with α-GalCer (100 ng/mL, 6 h). Afterward, we washed APCs and co-cultured the αGC-charged APCs (C1R-CD1d or LX2 cells) with human primary iNKT cells for 24 h, and IFNγ was determined in the extracellular media by enzyme-linked immunosorbent assay (ELISA) **(C,D)**, APCs (LX2 and C1R-CD1d) were treated with α-GalCer (100 ng/mL, 6 h). Afterward, we washed APCs and co-cultured the α-GalCer-charged APCs with human primary iNKT cells in the presence or absence of CTSB or CTSS inhibitors for 24 h, and IFNγ was determined in the extracellular media by ELISA. Results are given as a mean ± SD of five independent experiments; *p < 0.001 vs Control and ^#^*p* < 0.001 vs α-GalCer alone.

iNKT cell activation follows essentially a two-step process. First, APCs load the antigen that binds to CD1d, and in the second step, iNKT cells recognize the antigen presented by CD1d that is expressed on the cell surface of APCs. To better evaluate the role of cysteine cathepsins in APC-mediated iNKT cell activation as APCs we used C1R-CD1d cells, a human lymphoblastoid cell line (C1R) stably transfected to express human CD1d (35). Additionally, we tested LX2 cells, a human HSC line that can also act as a non-professional APC in the liver (44) to validate if the results obtained in the C1R-CD1d cell line could be translated to human hepatic cells. In the first approach, to evaluate the effect of cysteine cathepsins on the loading of antigen to CD1d, we incubated α-GalCer with APCs for 6 h in the presence or absence of CTSB or CTSS inhibitors. Afterward, we washed the APCs and co-cultured the α-GalCer-charged APCs, C1R-CD1d or LX2 cells, with human primary iNKT cells for 24 h. iNKT cell activation was measured by detecting IFNγ in the extracellular media. As shown in Figures 5A,B, α-GalCer loaded APCs induced iNKT cell activation, which was greater for C1R-CD1d cells than in LX2 cells. This response was significantly diminished when either APC cell type was pre-incubated with CTSS inhibitor, but not with CTSB inhibitor, consistent with our previous observations *in vivo*. This suggests that CTSS, but not CTSB, participates in the lysosomal loading of antigen onto CD1d in APCs. Additionally, we tried to specifically silence CTSB and CTSS. Unfortunately, the reduction in CTSB or CTSS protein levels was compensated by simultaneous increases by other cysteine cathepsins (data not shown), as previously detected in LX2 cells (3).

Next we evaluated the role of cysteine cathepsins only in the generation of cytokines by iNKT cells that have been activated after TCR-mediated recognition of antigen-bound to CD1d in APCs. To this aim, APCs were pre-loaded with α-GalCer for 6 h, and afterward cysteine cathepsin inhibitors were added along with primary human iNKT cells for 24 h. In this setting, both CTSB and CTSS inhibitors were able to reduce IFNγ release (Figures 5C,D). Thus, the results from these experiments suggest that CTSS, but not CTSB, interferes with antigen loading and traffic to bind CD1d, while both cysteine cathepsins alter IFNγ production once iNKT cells have been activated by APCs.

### CTSS, but Not CTSB, Reduces NPC2 and Enhances Ii-p10 Levels in APCs

**Figure 6:**
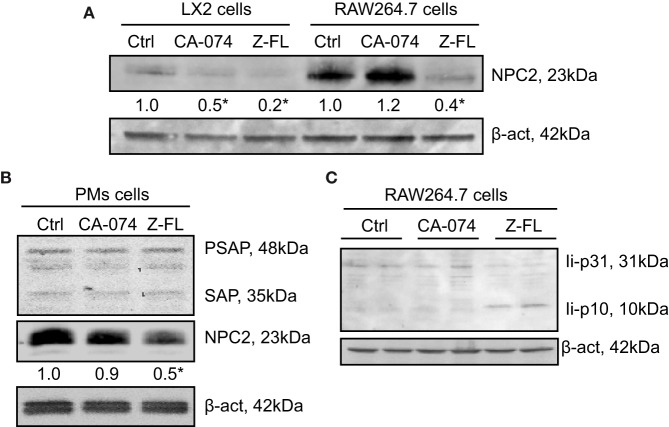
Effect of Cathepsin B (CTSB) or Cathepsin S (CTSS) inhibition on Niemann-Pick 2 protein (NPC2) and saposin protein expression. **(A,B)** LX2, RAW264.7 cells, and primary peritoneal macrophages (PMs) were incubated with CTSB (CA-074, 25 µM in LX2 cells, 75 µM in RAW264.7 and PMs, o/n) or CTSS inhibitors (Z-FL, 7.5 µM, o/n in LX2 cells, and for 4 h in RAW264.7 and PMs). **(C)** RAW264.7 cells were treated with CTSB and CTSS inhibitors (CA-074, 75 µM; and Z-FL, 7.5 µM) for 4 h, and protein expression was determined afterward. Representative immunoblots of three independent experiments. NPC2 levels were quantified as compared to β-actin (*n* = 3) *P < 0.05 vs. control cells.

Given that cysteine cathepsin inhibition, especially CTSS, reduced iNKT activation after α-GalCer challenge, we evaluated the effect of CTSS and CTSB inhibitors on the status of proteins related to antigen uptake and traffic to lysosomes. Several proteins have been described as responsible for the lysosomal lipid loading into CD1d, among them are the Niemann-Pick 2 protein (NPC2) (45–47) and the saposins (48, 49). Therefore, we first analyzed if expression of NPC2 and saposins were affected by cathepsin inhibition in APCs. As APCs, we analyzed LX2, RAW264.7 cells, and mouse PMs. Proteins levels of NPC2 in LX2 and RAW264.7 cells after exposure to CTSB or CTSS inhibitors were analyzed (Figure 6A). As shown, and consistent with our previous results, CTSB did not significantly affect expression of these proteins in all cell types analyzed. However, CTSS inhibition resulted in a consistent decreased in NPC2 expression, which could account for the deficient iNKT cell activation observed. Saposins were not detected by immunoblot in either LX2 or RAW264.7 cells. In contrast, saposins were detected and remained unchanged upon cathepsin B or S inhibition in primary PMs, although NPC2 decreased after CTSS inhibition (Figure 6B). As stated in the introduction, the association of invariant (Ii) chain with MHC class II dimers is required for proper antigen presentation to CD4^+^ T cells by APCs, and CTSS has been involved in the late stage processing of Ii-p10 (25–29). Thus, we analyzed in RAW264.7 cells whether CTSS inhibition could hamper antigen presentation by blocking Ii-p10 processing. As shown in Figure 6C, CTSS but not CTSB inhibition resulted in defective processing of Ii-p10, fragment that accumulated only in CTSS inhibitor treated RAW264.7 cells. These results indicate that CTSS could be implicated both in endosomal lipid loading and in CD1d trafficking.

## Discussion

iNKT cells are lymphocytes sharing characteristics of both innate and adaptive immunity, having a function in bridging the two types of immune responses. iNKT cells are highly associated with early inflammatory responses and they have a prominent influence on a wide range of immune and inflammatory situations. The murine liver is highly enriched in iNKT cells, and in order to stimulate iNKT cells, the α-GalCer antigen is more effective if it is internalized for loading onto CD1d in lysosomes (50, 51). However, the frequency of iNKT cells in the human liver is much lower, so mouse models addressing immune mediated liver damage must be discussed carefully when trying to interpret their importance for human disease (52).

The nature of NKT cells in the liver is quite controversial. NKT are believed to have pro-inflammatory properties given that they secrete IFNγ and can kill hepatoyctes by the release of FasL. In the context of liver pathologies, iNKT cell activation aggravates Hepatitis B Viral infection, primary biliary cirrhosis and NASH in experimental mouse models. However, NKT cells can also act as helper cells to facilitate Tregs expansion and suppress autoimmunity (20, 52). In fact, in patients with autoimmune hepatitis the number of iNKT cells is reduced, contributing to disease progression (53). This ambiguous behavior of iNKT cells may be explained, in part, by the opposed roles of iNKT-derived cytokines IL-4 and IFNγ (54). Injection of α-Galcer into mice induces iNKT activation, with rapid production of IL-4 but delayed production of IFN-γ, which results in mild hepatitis and liver injury (54), as we have also observed. In contrast, iNKT cells activation by α-GalCer has been shown to inhibit hepatitis viral replication and liver cancer growth in animals. This last observation has results in the testing of α-GalCer in clinical trials for these diseases. Of note, while α-GalCer was reported to be well tolerated few beneficial effects were seen in patients (54).

IL-12 production by APCs can activate iNKT cells independent of the TCR (37). IL-12 also can augment the TCR-induced response to weaker antigens. Activation of iNKT cells is response to bacterial infection is predominantly mediated by KCs (41). In the liver, LPS and the LPS-induced cytokine TNF play a central role in liver homeostasis and inflammation through the activation of the NF-κB transcription factor in different liver cell populations (55). LPS is a potent inducer of liver damage, while iNKT cell population expands after LPS challenge, as we have observed, being this increase blocked by the use of CTSB and CTSS inhibitors. In addition, the CTSS inhibitor not only blocked iNKT expansion after LPS challenge but also the resident iNKT population was reduced as compared to control mice. This is consistent with the observation that CTSS^−/−^ mice have a slight decreased number of iNKT cells (32). Of interest, the lack of expansion of iNKT cells in response to LPS, observed *in vivo* after CTSB or CTSS inhibition, could be due to reduced IL-12 generation by APCs, since LPS-exposed KCs generate IL-12 and CTSB and CTSS inhibitors decreased this induction. This hypothesis is consistent with a role for cysteine cathepsins in the activation of NF-κB in APCs, as we have recently described for several cell types, among them primary hepatic cells, such as hepatocytes, HSCs, and KCs, and in different cell lines such as Hep3B cells (a human hepatoma cell line), LX2 cells (human HSCs), and murine RAW264.7 macrophages (3). Although we cannot state that this regulation of CTSB and CTSS on NF-κB activation is universal, all the cell types we have analyzed so far have behaved in the same way. Therefore, it would not be surprising if the activation of NF-κB cells in iNKT cells followed the same pattern, which rests to be tested. Our *in vitro* experiments suggest that both CTSB and CTSS are important for IFNγ secretion, once iNKT cells are activated. Although additional studies will be required to better understand this observation, we venture that this could be related to the activation of NF-κB, which is necessary for IFNγ gene transcription (56, 57). Multiple signaling pathways including the TLRs, TCR, CD28, and IL-18 receptors converge on the NF-κB family of transcription factors. The five NF-κB family members include RelA (p65), RelB, and c-Rel, which can transactivate target genes when dimerized, and NF-κB1 (p50) and NF-κB2 (p52), which do not contain transactivation domains and on their own can repress activation (58). In addition, little is known about which NF-κB heterodimers drive IFNγ generation in iNKT cells. It is important to acknowledge that in CTSS knockout mice an impaired production of IFNγ and TNF has been described (31), observation in accordance with our results. We could corroborate in KCs and in RAW264.7 macrophages that CTSB and CTSS inhibition decreased NF-κB activation after LPS challenge by detecting p65-NF-κB protein expression in nuclear extracts. Since IL-12 generated after LPS-stimulation in macrophages relies on NF-κB activation, and CTSB and CTSS inhibitors decreased not only IL-12 generation in KCs but also IFNγ secretion in co-culture experiments with KCs and iNKT, our study indicates that the lack of iNKT expansion observed after treatment with CTSB and CTSS inhibitors after LPS administration *in vivo* could be due to reduced NF-κB-dependent IL-12 generation by KCs.

Next, we stimulated iNKT cells with α-GalCer to induce hepatic liver damage and we analyzed production of IL-4 and IFNγ, which were only decreased after CTSS inhibition. To better understand the role of each of the cysteine cathepsins, we carried out *in vitro* assays to dissect the potential participation of CTSB and CTSS in iNKT cell activation. Our co-culture studies using human APCs cell lines and primary human iNKT indicate that CTSS, which has been associated with antigen presentation by MHC I and MHC II (6, 59, 60), is essential for correct CD1d-loading with specific lipid antigens in APCs. This is consistent with the fact that although widely expressed, some cysteine proteases are found in significantly greater quantities in certain cell types, and in particular, while CTSB has a more ubiquitous expression; CTSS is preferentially expressed in APCs, including DCs, macrophages, and B cells (61). The participation of CTSS in endosomal antigen loading of MHC class II proteins, by cleavage of Class II-associated Ii-p10 to CLIP, has been previously established (5, 24–29). This cleavage also has been implicated in CD1d-mediated antigen presentation (30). In this context, we have validated that CTSS, but not CTSB, inhibition results in Ii-p10 accumulation in macrophages. We additionally show that CTSS inhibition contributes to the degradation of NPC2 protein implicated in lipid antigen loading (46, 47) in APCs. NPC2 has been reported to affect the transport and loading of glycolipid onto CD1d in thymocytes of NPC2-deficient mice. These mice display an impaired thymic selection of Valpha14 natural killer T cells (NKT cells) and a subsequent reduction of NKT cells in the periphery. In addition, thymocytes and splenocytes from NPC2-deficient mice were poor presenters of endogenous and exogenous lipids to CD1d-restricted Valpha14 hybridoma cells, effect that was corrected when administering recombinant NPC2 (46). Thus, we can state that the role of NPC2 is not liver specific, since in addition we have observed this same effect in LX2 cells, RAW264.7 cells, and primary PMs. NPC2 depletion rends APCs unable to correctly load α-GalCer onto CD1d. Therefore, CTSS appears to indirectly contribute to both lipid loading, by maintaining NPC2, and to CD1d-mediated antigen presentation, by converting Ii-p10 to CLIP. However, the fact that CTSS inhibition results in NPC2 degradation appears not be a CTSS direct effect, given the proteolytic nature of CTSS. Most likely NPC2 degradation is the consequence of the altered endosomal architecture observed upon CTSS inhibition and Ii-p10 accumulation (25, 28).

Cathepsin B (CTSB) and CTSS inhibition reduced iNKT cell activation and expansion after LPS, apparently by interfering with NF-κB-dependent IL-12 expression in APCs, resulting in iNKT cell activation. Conversely, after α-GalCer administration the main mechanism responsible for iNKT cell activation is CD1d-loading of α-GalCer in APCs, where CTSB is not involved, but CTSS seems essential, according to our studies. Moreover, according to the enhanced number of iNKT that stain positive for Annexin V staining after CTSS inhibition, it appears that the two inhibitors act differently on iNKT cells *in vivo*. Thus, to better evaluate the effect of the inhibitors on the number of iNKT cells under these conditions and to avoid loss of CD1d-tetramer staining due to TCR downregulation, further studies, such as analyzing the Vα14-Jα18 rearrangement, will be required.

Of interest, in the LPS study CTSB inhibition was more effective than CTSS inhibition in reducing liver damage in the LPS model, despite the fact that the CTSS inhibitor was better in terms of preventing iNKT expansion. This observation could be explained by the acknowledged participation of CTSB in TNF-dependent hepatocyte apoptosis and liver damage (39, 62, 63). Liver damaged, as measured by ALT transaminases, is a read-out of hepatocyte damage, being hepatocytes the predominant hepatic cell type by far. In addition, LPS mediated liver injury in TNF- but not FasL dependent (64), and it has been described that caspase-mediated release of CTSB from lysosomes enhances mitochondrial release of cytochrome *c* and subsequent caspase activation in TNF-treated hepatocytes (39, 63). Of note, CTSB inhibition or CTSB knockout render mice resistant to TNF-mediated liver injury and hepatocyte apoptosis (39, 63) which agrees with our observations. However, as described by others (62), CTSB does not participate in FasL-mediated hepatocyte apoptosis, since it does not depend on caspase-mediated lysosomal permeabilization (62). This last observation could explain why in the α-GalCer model, where TNF is involved in α-GalCer-induced upregulation of FasL on NKT cells, but hepatocyte damage depends mainly on FasL (65), CTSB inhibition did not protect against α-GalCer mediated hepatitis.

We did not observe any effect of CTSB inhibitor in cytokine secretion by iNKTs *in vivo* after α-GalCer, which clearly contradicts our *in vitro* studies where CA-074 was very effective in this cell type. A probable explanation for this discrepancy is an insufficient CTSB inhibition in all non-parenchymal cell types *in vivo* to block the potent α-GalCer activation pathway. Differences in CTSB activity exists among cell types which can explain the differences observed *in vitro* and *in vivo*. In fact, our previous study reported that RAW264.7 macrophages, and KCs have higher CTSB activity, more than 300%, as compared to hepatic parenchymal cell types, Hep3B or primary hepatocytes (3), and that 75 µM CA-074 CTSB inhibitor was necessary to completely abolish CTSB activity in macrophages, while only 25 µM was needed for hepatocyte CTSB inhibition. The abundant nature of CTSB makes difficult its complete inhibition *in vivo*, but not *in vitro*.

**Figure 7:**
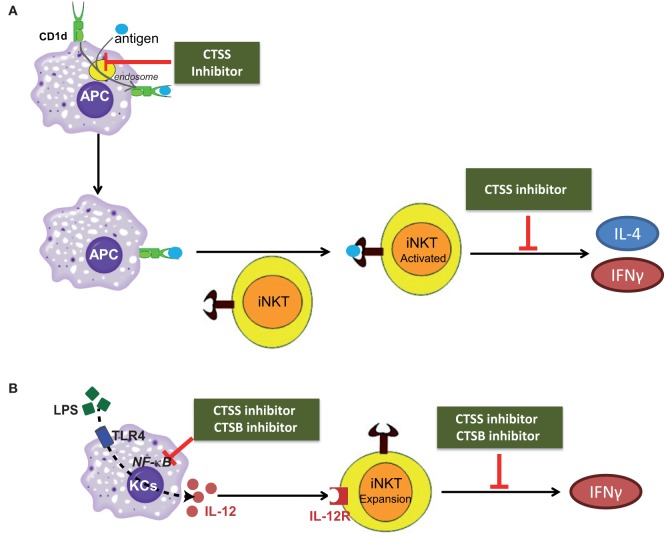
Scheme depicting the role of cathepsin B (CTSB) and cathepsin S (CTSS) in the direct, or antigen mediated; and indirect, or IL-12 mediated, iNKT cell activation. **(A)** CTSS participates both in APC-loading of lipid antigen to CD1d in the endosome and in cytokine secretion from activated iNKTs. **(B)** CTSB and CTSS inhibition reduced IL-12 production by TLR4-stimulated Kupffer cells (KCs), by diminishing NF-κB activation, resulting in reduced iNKT cell expansion after LPS exposure.

To summarize our results, cysteine cathepsins CTSB and CTSS participate in the direct, or antigen mediated; and indirect, or IL-12 mediated, activation of iNKT cells as illustrated in the scheme in Figures 7A,B. In conclusion, our study discloses the differential role of cysteine cathepsins, CTSB and CTSS, in the activation of iNKT cells.

## Ethics Statement

All animals received humane care according to the criteria outlined in the “Guide for the Care and Use of Laboratory Animals.” Animal procedures were approved by the Animal Experimentation Ethics Committee (CEEA) from the Universitat de Barcelona. Human iNKT cell lines were generated using human blood. Informed consent was obtained from all subjects. Study was approved by La Jolla Institute for Allergy and Immunology Institutional Review Board.

## Author Contributions

AP, EG, SC, and MM performed the experiments; AC, AM, MK, and MM designed experiments, provided materials and funding. MM was primarily responsible for writing the manuscript. All authors contributed to manuscript editing and approval.

## Conflict of Interest Statement

The authors declare that the research was conducted in the absence of any commercial or financial relationships that could be construed as a potential conflict of interest.
